# Comparative Study on the Performance and Hydration Mechanism of Coal Gangue Cementitious Materials with Different Alkali Activators

**DOI:** 10.3390/ma19081631

**Published:** 2026-04-18

**Authors:** Chao Geng, Yajie Gao, Quanming Li, Zongyuan Mao, Xianfeng Shi, Wei Li, Yajie Wang, Cheng Chen, Hong Zhang, Yukai Wang

**Affiliations:** 1 School of Civil Engineering, North China University of Technology, Beijing 100144, China; gengchao@ncut.edu.cn (C.G.); liweiustb2021@163.com (W.L.); w.angyajie@foxmail.com (Y.W.); 002420@ncut.edu.cn (C.C.); wangyukai@ncut.edu.cn (Y.W.); 2 China Academy of Building Research, Beijing 100013, China; 3 China Building Technique Group Co., Ltd., Beijing 100020, China; 4 School of Mine Safety, University of Emergency Management, Langfang 065201, China; 5 Key Laboratory of Disaster Prevention and Control Technology and Equipment for Tailings Ponds, State Administration of Mining Safety, Beijing 100038, China; i008_zhanghong@126.com; 6 State Key Laboratory of Hydroscience and Engineering, Tsinghua University, Beijing 100084, China

**Keywords:** CG, alkali activator, cementitious properties, N-A-S-H gel, hydration mechanism, resource utilization

## Abstract

Coal gangue (CG) ranks among China’s most significant industrial solid by-products. In response to China’s carbon neutrality commitments and the growing emphasis on resource recycling, finding effective ways to valorize CG has emerged as a pressing concern. Based on the mineral composition and chemical composition characteristics of CG, this study systematically investigated the enhancement effects of three alkali activators (Na_2_SiO_3_, NaOH, and Ca(OH)_2_) on the cementitious properties of CG. Through different dosage and compressive strength tests, the efficiency ranking of the three activators was determined as follows: Na_2_SiO_3_ > Ca(OH)_2_ > NaOH. A 10% Na_2_SiO_3_ dosage combined with 28-day curing was identified as the optimal condition for achieving sufficient reaction and structural densification. Under these conditions, the compressive strength of CG cementitious material reached 6.4 MPa, representing an increase of 190.9% compared to the blank group (2.2 MPa), significantly superior to Ca(OH)_2_ (69.55%) and NaOH (62.27%). X-ray diffraction (XRD) and scanning electron microscopy-energy dispersive spectroscopy (SEM-EDS) analyses revealed that alkali activators function primarily by disrupting the crystalline framework of CG, promoting the cross-linking polymerization of silicon–aluminum monomers to generate dense cementitious products, thereby improving material performance. The Na_2_SiO_3_ is attributed to its “dual activation effect”, providing OH^−^ to create an alkaline environment while supplying reactive silicate ions (SiO_3_^2−^) to accelerate N-A-S-H gel and C-A-S-H gel formation. These findings offer guidance for optimizing CG-based cementitious formulations for formula optimization and large-scale utilization of CG cementitious materials.

## 1. Introduction

Coal continues to dominate China’s energy mix; it accounted for 66.6% of the energy production structure in 2023. By the end of 2023, China’s coal reserves reached 218.57 billion tons, and raw coal production reached 4.76 billion tons in 2024, setting a historical record [1]. Intensive coal extraction has led to steadily rising CG output, showing an overall upward trend from 2011 to 2022, mainly distributed in major coal-producing areas such as Shanxi, Inner Mongolia, and Shaanxi provinces. Currently, China has formed more than 2700 gangue mountains, with a total stockpile exceeding 7 billion tons, occupying more than 200,000 mu of land, establishing CG as a major contributor to China’s industrial waste burden [2,3]. During the stockpiling process, the heavy metals and acidic substances contained in CG can easily infiltrate underground through rainwater erosion, polluting water resources and soil. Meanwhile, the loose mountain structure poses collapse risks, threatening the surrounding ecological environment and human safety [4].

To promote the resource utilization of CG, China has successively issued policies such as the “Administrative Measures for Comprehensive Utilization of CG” and “Opinions on Strengthening Clean and Efficient Utilization of Coal”, explicitly proposing to advance CG utilization in ecological restoration, backfilling mining, and engineering construction. CG is rich in minerals such as kaolinite and quartz, as well as chemical components like Al_2_O_3_ and SiO_2_, possessing the potential for transformation into resource products such as cementitious materials and adsorbent materials [5,6]. However, its low natural reactivity limits high-value utilization efficiency, making activation technology to enhance its performance a key research direction [7,8,9,10].

Alkali-activated materials (AAMs) are novel cementitious materials prepared using industrial solid wastes as raw materials and alkaline solutions as activators, with advantages of low carbon footprint, environmental friendliness, and excellent mechanical properties [11,12]. The alkali activation process is generally understood to proceed through dissolution of Si-O and Al-O bonds in the precursor under high-pH conditions, followed by condensation polymerization of dissolved silicate and aluminate species into gel networks—predominantly N-A-S-H or C-A-S-H gels depending on the Ca content of the system [13]. Common alkali activators include NaOH, Ca(OH)_2_, and Na_2_SiO_3_, each imparting distinct activation pathways. NaOH provides strong alkalinity that promotes rapid dissolution of Si-Al phases in the precursor; however, the absence of an external silicon source limits the density of the resulting gel network [14]. Ca(OH)_2_ supplies Ca^2+^ ions that favor C-A-S-H gel formation and long-term strength development, but its relatively low solubility restricts early-age reactivity [15,16,17,18]. Na_2_SiO_3_ simultaneously provides OH^−^ for alkaline activation and SiO_3_^2−^ as an additional reactive silicon source, which accelerates gel polycondensation and promotes denser microstructures; the silicate modulus has been identified as a critical parameter governing gel product composition and mechanical performance [7].

Regarding CG-based AAMs specifically, Wang et al. [8] demonstrated that activator type is a decisive factor controlling the mechanical behavior of CG-based alkali-activated systems. Feng et al. [10] explored the alkali activation of spontaneous combustion CG, emphasizing the importance of raw material characteristics on cementitious performance. Zhang et al. [9] reported that thermal pre-treatment of CG significantly enhances its reactivity toward alkali activation by converting crystalline kaolinite to amorphous metakaolinite. However, the majority of existing studies have focused on CG–slag or CG–fly ash blended systems [14], in which the contributions of supplementary cementitious materials and activator chemistry are inherently coupled and difficult to separate. Systematic comparative studies of CG systems—without supplementary materials—under identical activator dosage conditions remain scarce. Furthermore, while the relative merits of individual activators have been discussed in blended systems, the mechanistic basis for performance differences among NaOH, Ca(OH)_2_, and Na_2_SiO_3_, specifically in CG precursor systems, at the phase evolution and microstructural level, has not been established.

To address the above issues, this study uses CG from Taiyuan, Shanxi as the sole precursor and systematically investigates the effects of three alkali activators—NaOH, Ca(OH)_2_, and Na_2_SiO_3_—at identical dosage levels (5%, 7%, 10%, and 12%) on the cementitious properties of CG. XRD phase analysis and SEM-EDS are employed to characterize phase evolution and microstructural development across activator types and curing ages. The specific objectives are as follows: (1) to determine the compressive strength development patterns and activation efficiency ranking of the three activators in a CG system; (2) to identify the phase assemblages and microstructural features associated with each activator type; (3) to provide practical guidance for optimizing alkali activator selection for large-scale CG valorization, such as mine backfill.

## 2. Materials and Methods

### 2.1. Experimental Materials

#### 2.1.1. Coal Gangue

**Figure 1:**
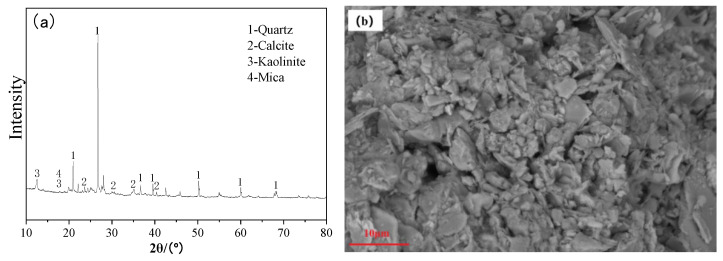
XRD pattern and micromorphology of CG: (**a**) XRD pattern; (**b**) SEM micrograph.

**Figure 2:**
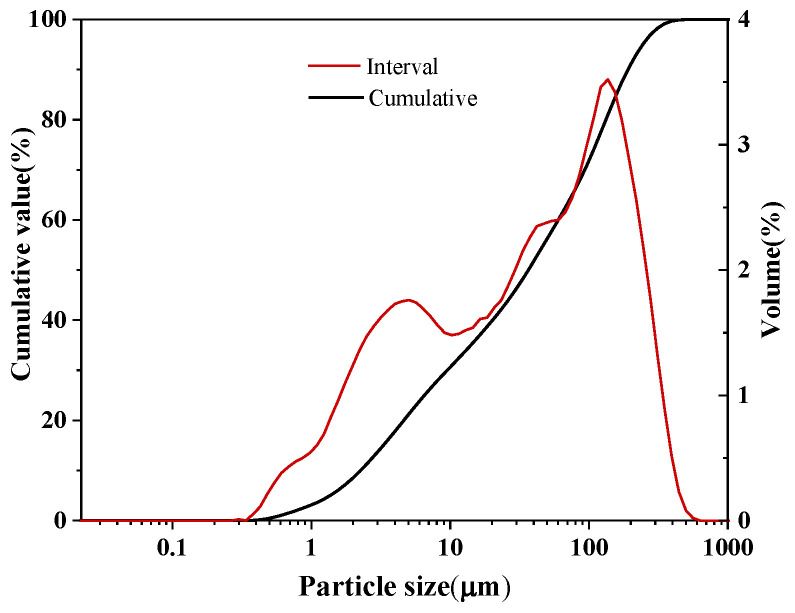
Particle size distribution curve of CG.

CG constitutes the lithic fraction discarded throughout coal extraction and processing operations, predominantly comprising mineral assemblages including kaolinite, mica, and quartz. The CG specimens investigated herein were sourced from a Taiyuan-based colliery, Shanxi, subjected to jaw crushing followed by ball milling for 2 h before sieving. The chemical composition of CG was determined by X-ray fluorescence (XRF) spectrometry using a PANalytical Axios spectrometer (Malvern Panalytical Ltd., Malvern, UK, as described in Section 2.3.2). The elemental oxide distribution is presented in Table 1. It can be seen that the main chemical components of CG are SiO_2_, Al_2_O_3_, and Fe_2_O_3_, with a silicon–aluminum ratio (SiO_2_/Al_2_O_3_) of 2.73. The XRD pattern shown in Figure 1a indicates that the main minerals in this CG are quartz, mica, calcite, and kaolinite, demonstrating its potential as a precursor for alkali-activated materials [15]. The micromorphology shown in Figure 1b reveals that CG consists of loose particles without agglomeration. Particle size distribution curve of CG shown in Figure 2.

#### 2.1.2. Activators

Three alkaline activators were employed: NaOH, Ca(OH)_2_, and Na_2_SiO_3_ solution. NaOH and Ca(OH)_2_ were analytical grade with 99% purity (Sinopharm Chemical Reagent Co., Ltd., Shanghai, China). Na_2_SiO_3_ had a modulus of 1.0 M, with Na_2_O content of 19.3% (industrial grade, Shandong Silicate Co., Ltd., Jinan, China).

### 2.2. Experimental Procedure and Design

**Figure 3:**
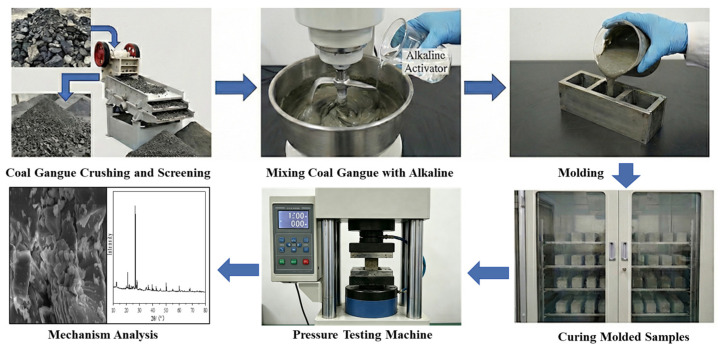
Experimental flow chart.

This study examined how different alkali activators influence CG cementitious performance. Three activators, Ca(OH)_2_, NaOH, and Na_2_SiO_3_, were tested at varying dosages. Compressive strength measurements were used to evaluate the activation efficiency. The experimental procedure and mix proportions are presented in Figure 3 and Table 2, respectively. The activator dosage refers to the mass percentage of the alkali activator relative to the mass of coal gangue. The water-to-binder (W/B) ratio refers to the ratio of the mass of water to the sum of the masses of the alkali activator and coal gangue.

### 2.3. Test Methods

#### 2.3.1. Particle Size Distribution Test

Laser particle size distribution tests were performed on CG using the dry method to avoid interference from water-soluble components. The test instrument was a Mastersizer 3000 laser particle size analyzer (Malvern Panalytical Ltd., Malvern, UK). An appropriate amount of powder was placed in the dry dispersion system, with dispersion pressure set at 0.2 MPa, 3 scans, using air as the dispersion medium, to determine the particle size distribution range (D10, D50, and D90) and distribution curves. This test aimed to characterize the particle gradation of CG raw material and assess its influence on the alkali-activation reaction rate and cementitious material performance.

#### 2.3.2. Multi-Element Analysis

Multi-element analysis of CG raw materials was performed. Chemical analysis was conducted via XRF spectrometry. The test instrument was a PANalytical Axios X-ray fluorescence spectrometer (Malvern Panalytical Ltd., Malvern, UK). In total, 5 g of powder was pressed into a 40 mm diameter disk sample using the boric acid edge pressing method (pressure 30 MPa; holding time 30 s). Detection parameters were set as reference [14]. Analyzed elements included major oxides and trace heavy metal oxides, aiming to clarify key chemical characteristics of raw materials such as silicon–aluminum ratio and calcium content, providing data support for revealing the material basis of alkali-activation reactions in blended systems.

#### 2.3.3. Preparation of Alkali-Activated CG Specimens

CG raw material, water, and alkali activator were sequentially added to the cement mortar mixer pot, followed by slow mixing for 180 s, pausing for 10 s, then fast mixing for 180 s. The mixed alkali-activated material was transferred into 40 mm cubic steel molds, followed by mechanical vibration (30 s) for void elimination. After smoothing the surface, specimens underwent controlled curing (>90% RH; 20 ± 1 °C) in a humidity chamber, with performance testing conducted after curing for 3 d, 7 d, and 28 d, respectively. Each group of experiments was performed in triplicate.

#### 2.3.4. Compressive Strength Test

Compressive strength was measured on a YAW-300B servo-controlled testing machine at a loading rate of 2.4 kN/s, with reference to the loading procedure specified in GB/T 17671-2021 [19]. The compressive strength (*R_c_*) was determined using Equation (1):

*R_c_* = F_c_/A (1)

where F_c_ represents the maximum load applied at failure of the 40 mm cubic specimen (N), and A represents the compressed area of the cube face (40 mm × 40 mm = 1600 mm^2^).

#### 2.3.5. XRD Analysis

Phase composition analysis of CG raw materials and specimens (ground into powder) was performed using a Bruker D8 Advance XRD. Parameters were adopted from prior work [14]. During analysis, the diffraction peak positions and intensities in the patterns were compared with standard phase databases (ICDD PDF-2 database) to determine the phases present in the samples.

#### 2.3.6. SEM Analysis

Specimens were tested using an FEI Quanta 650 SEM coupled with a Bruker QUANTAX EDS spectrometer. Dried samples were cut into small pieces to form fresh fracture surfaces of approximately 0.5–1 cm^2^, gold-sputtered, and then observed under the SEM combined with EDS analysis.

## 3. Results and Discussion

### 3.1. Compressive Strength Analysis

**Figure 4:**
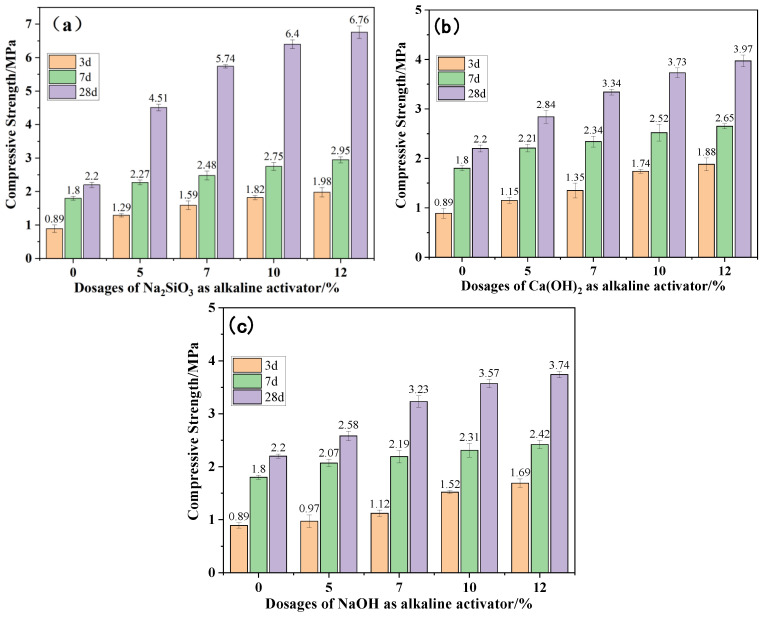
Effects of different alkali activators and dosages on compressive strength: (**a**) Na_2_SiO_3_; (**b**) Ca(OH)_2_; (**c**) NaOH.

The compressive strength development patterns of CG cementitious materials with different alkali activators and different dosages are shown in Figure 4a–c. Each group was tested in triplicate, and the results are presented as mean values with standard deviation.

As shown in the figures, under the action of three different alkali activators, compressive strength exhibited a positive correlation with activator concentration. When the specimens were cured for 28 d, the strength without an alkali activator was 2.2 MPa. When the activator dosages were 5%, 7%, 10%, and 12%, the compressive strengths corresponding to Na_2_SiO_3_ were 4.51, 5.74, 6.4, and 6.76 MPa, with growth rates of 105.0%, 160.9%, 190.9%, and 207.3%, respectively; the compressive strengths corresponding to Ca(OH)_2_ were 2.84, 3.34, 3.73, and 3.97 MPa, with growth rates of 29.09%, 51.82%, 69.55%, and 80.45%, respectively; the compressive strengths corresponding to NaOH were 2.58, 3.23, 3.57, and 3.74 MPa, with growth rates of 17.27%, 46.82%, 62.27%, and 70.00%, respectively. The above results indicate the three activators exhibited markedly different activation capacities on CG, with efficiency ranking as follows: Na_2_SiO_3_ > Ca(OH)_2_ > NaOH. To further verify the differences in activation efficiency among the three activators, phase composition and microstructural analyses are presented in Section 3.2.

At the same activator dosage, Na_2_SiO_3_ shows the most significant activation effect. At 10% activator dosage, the compressive strengths at 3 d, 7 d, and 28 d curing were 1.47, 1.84, and 1.72 times those of Ca(OH)_2_, and 1.20, 1.19, and 1.79 times those of NaOH, respectively. Therefore, the advantage of Na_2_SiO_3_ is more significant at the same dosage. This is mainly because the hydrolysis of Na_2_SiO_3_ enhances the cementitious properties of CG through dual pathways: in addition to the OH^−^ generated enhancing system alkalinity to promote dissociation of silicon–aluminum phases in the CG system and release of active components, it also provides silicate ions (SiO_3_^2−^) as an external silicon source, which combines with dissolved aluminum species and sodium ions, forming dense cementitious structures dominated by amorphous hydrated sodium aluminosilicate (N-A-S-H) gel, as evidenced by the broad diffuse humps in XRD patterns rather than crystalline diffraction peaks through condensation reactions [20,21].

The formation of cementitious gel products in alkali-activated CG systems can be understood from a molecular chemistry perspective. Under alkaline conditions, OH^−^ ions attack and cleave the Si-O-Si and Si-O-Al bonds in the CG mineral framework, releasing soluble silicon and aluminum monomers according to the following reactions:

Si-O-Si + OH^−^ → H_3_SiO_4_^−^/H_2_SiO_4_^2−^ (2)

Al-O-Al + OH^−^ → Al(OH)_4_^−^ (3)

The released monomers subsequently undergo condensation polymerization. In the presence of Na^+^, aluminate and silicate ions cross-link to form amorphous N-A-S-H gel:

Na^+^ + Al(OH)_4_^−^ + H_3_SiO_4_^−^ → N-A-S-H gel (4)

In systems where Ca^2+^ is available (Ca(OH)_2_, the activator or calcium released from CG), C-A-S-H gel forms through the following reaction:

Ca^2+^ + H_3_SiO_4_^−^ + Al(OH)_4_^−^ → C-A-S-H gel (5)

In the sodium silicate system, the additional supply of SiO_3_^2−^ ions further promotes reactions Equations (4) and (5), accelerating gel network formation and contributing to the superior cementitious performance observed.

To further verify the action mechanisms of the three activators, phase and microstructure analyses were performed.

### 3.2. Hydration Reaction Mechanism Analysis

#### 3.2.1. Phase Composition Analysis

**Figure 5:**
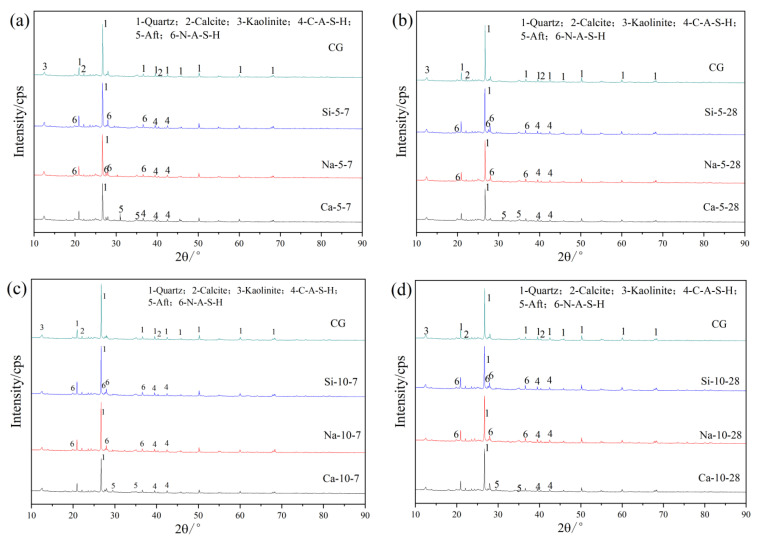
XRD patterns of CG with different alkali activators and dosages: (**a**) 5% dosage, 7 d curing; (**b**) 5% dosage, 28 d curing; (**c**) 10% dosage, 7 d curing; (**d**) 10% dosage, 28 d curing.

Figure 5a,b show the XRD patterns of Na_2_SiO_3_, Ca(OH)_2_, and NaOH at 5% dosage after 7 d and 28 d curing; Figure 5c,d show the XRD patterns of each activator at 10% dosage after 7 d and 28 d curing. First, the CG samples mainly contain mineral phases such as quartz, kaolinite, calcite, and mica. After alkali activation, phase changes occurred in all systems.

As can be seen from the figures, following alkaline treatment, quartz diffraction intensities diminished universally. Moreover, after activation of CG with Na_2_SiO_3_ and NaOH, broad amorphous humps characteristic of N-A-S-H and C-A-S-H gels were observed in the 2θ = 10–35° region, consistent with their known amorphous to semi-crystalline nature. These gel phases do not produce sharp diffraction peaks but are identified by the broadened diffuse scattering features, which intensified with increasing activator dosage, indicating greater gel formation. After activation with Ca(OH)_2_, AFt and amorphous C-A-S-H gel were observed. With increasing activator dosage, the diffraction peaks of cementitious products significantly increased. At 10% dosage, activation product peaks were stronger, original mineral (quartz and kaolinite) peaks were weaker, reactions were more complete, and amorphous gel formation (N-A-S-H, C-A-S-H) and AFt precipitation (Ca(OH)_2_ activator) were more pronounced. According to Figure 5a–d, different activator types affect product phases: Na_2_SiO_3_ and NaOH systems are dominated by N-A-S-H and C-A-S-H, exhibiting persistent accumulation over extended durations; Ca(OH)_2_ activator, due to calcium ions, shows persistent prominent AFt peaks and enhanced C-A-S-H, reflecting its long-term contribution to ettringite and silicate gel [22]. Na_2_SiO_3_-activated samples exhibit the most prominent broad diffuse humps in the 2θ = 10–40° range, consistent with a greater extent of amorphous N-A-S-H gel formation [23,24,25] (Si-5-7, Si-5-28, Si-10-7, Si-10-28), with peak intensity significantly increasing with dosage, corresponding to the 28 d compressive strength growth rate of 207.3%, highly consistent with macroscopic mechanical properties. Therefore, the phase evolution results demonstrate that Na_2_SiO_3_ hydrolysis provides both an alkaline environment and an additional reactive silicon source, efficiently activating active silicon–aluminum components in CG and generating large amounts of amorphous cementitious products. In contrast, NaOH and Ca(OH)_2_-activated samples show relatively lower intensity of the amorphous hump in the XRD patterns, with inert phases (such as quartz) dominating, relying only on alkaline activation without active silicon supplementation, resulting in limited 28 d strength increases of 70.0% and 80.45%, respectively. This shows that Na_2_SiO_3_ has a dual activation effect, with its phase evolution highly consistent with macroscopic strength data, showing significant advantages in promoting cementitious product formation and improving later-age strength, while NaOH and Ca(OH)_2_ have weaker effects due to their single activation mechanism relying only on alkaline activation. Ca(OH)_2_ activator favors long-term AFt presence, while Na_2_SiO_3_ and NaOH activators dominate silicate gel accumulation, illustrating the long-term mineral phase evolution and activator regulation effects of alkali-activated materials.

#### 3.2.2. Microstructure Analysis

##### Activation of CG Cementitious Properties by Na_2_SiO_3_

**Figure 6:**
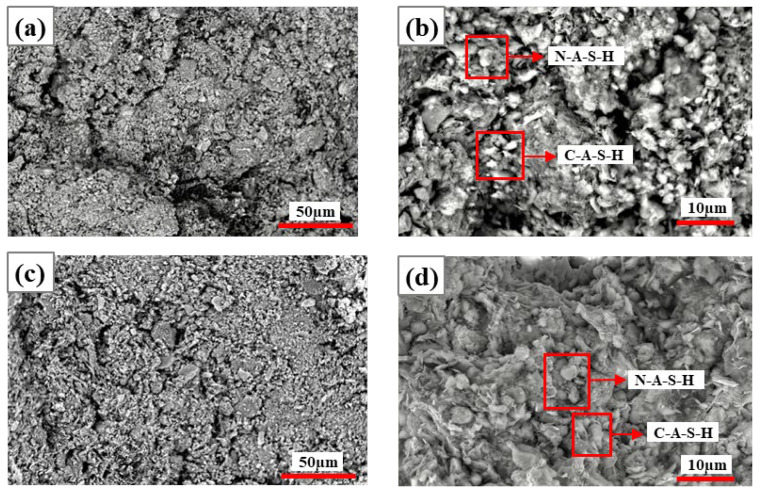
SEM images of specimens with 5% Na_2_SiO_3_ at 7 d and 28 d curing: (**a**,**b**) 7 d curing; (**c**,**d**) 28 d curing.

**Figure 7:**
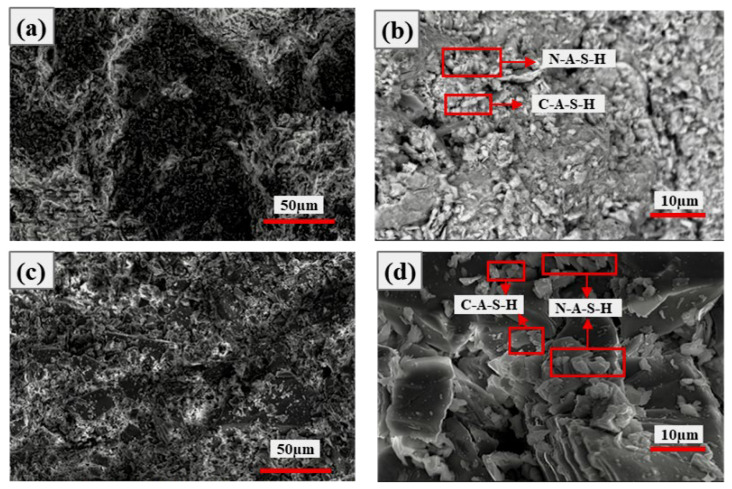
SEM images of specimens with 10% Na_2_SiO_3_ at 7 d and 28 d curing: (**a**,**b**) 7 d curing; (**c**,**d**) 28 d curing.

Figure 6 and Figure 7 show the SEM images of specimens at 5% and 10% Na_2_SiO_3_ dosage after 7 d and 28 d curing, respectively. Table 3 shows the elemental composition derived from the EDS spectra in Figure 6. As can be seen from Figure 6, at 5% Na_2_SiO_3_ dosage, CG hydration remained incomplete, yielding a porous microstructure, numerous cracks, and many unreacted particles. Extending curing time from 7 d to 28 d had a limited effect on performance improvement, indicating that low-dosage Na_2_SiO_3_ failed to effectively promote the formation of dense gel networks. At 28 d curing, C-A-S-H gel content slightly increased but was still insufficient to significantly improve structural compactness (Figure 6c,d).

In contrast, specimens at 10% Na_2_SiO_3_ dosage showed significantly enhanced compactness, reduced voids, and a tight structure compared to 5% specimens with extended curing time. At 7 d curing (Figure 7a,b), N-A-S-H gel constituted the predominant hydration phase, with small amounts of C-A-S-H gel formed in local areas. At 28 d curing in later hydration reactions (Figure 7c,d), N-A-S-H and C-A-S-H became the dominant phases, calcium content significantly increased (15–19%), Si/Al ratio stabilized at 6.0–8.0, and microstructural densification was markedly enhanced. Furthermore, Figure 7d shows that the two gels, N-A-S-H and C-A-S-H, are interconnected, indicating that they do not simply stack inside the solidified body but interact to jointly promote polymerization reactions in the system. This synergistic effect significantly improves solidification efficiency, thereby enhancing the mechanical properties of materials [26]. Combining Table 4, Na_2_SiO_3_ dosage has a significant influence on CG alkali-activated cementitious materialization reactions.

##### Activation of CG Cementitious Properties by Ca(OH)_2_

**Figure 8:**
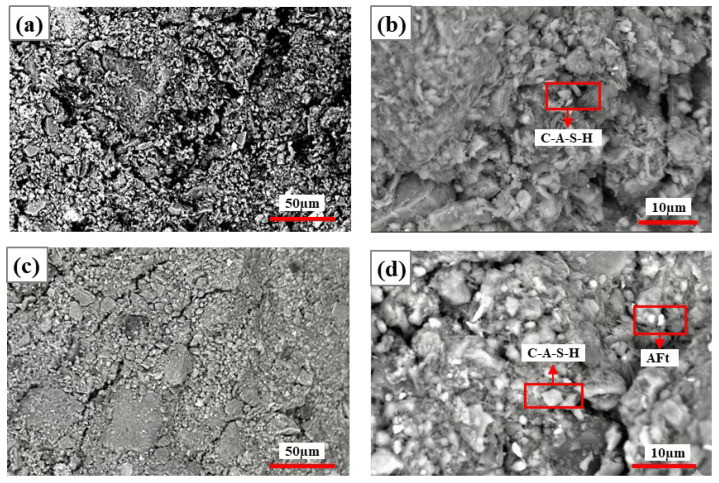
SEM images of specimens with 5% Ca(OH)_2_ at 7 d and 28 d curing: (**a**,**b**) 7 d curing; (**c**,**d**) 28 d curing.

**Figure 9:**
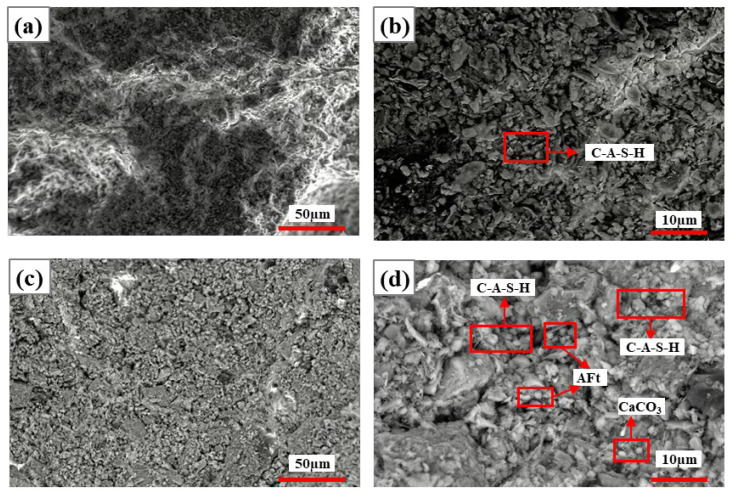
SEM images of specimens with 10% Ca(OH)_2_ at 7 d and 28 d curing: (**a**,**b**) 7 d curing; (**c**,**d**) 28 d curing.

Figure 8 and Figure 9 show the SEM images of specimens at 5% and 10% Ca(OH)_2_ dosage after 7 d and 28 d curing, respectively. As can be seen from Figure 8, at 5% Ca(OH)_2_ dosage, the system structure was loose in early hydration (Figure 8a,b), with few hydration products, much residual Ca(OH)_2_, small amounts of C-A-S-H gel, and limited aluminosilicate products. At 28 d curing (Figure 8c,d), the structure was slightly denser, hydration products slightly increased, C-A-S-H gel was more typical, and AFt also formed, but overall reaction was slow, and according to EDS spectra (Table 5), aluminum phase participation was limited.

As can be seen from Figure 9, at 10% Ca(OH)_2_ dosage, early hydration showed loose structure, poor uniformity, and few hydration products. With curing time extended to 28 d, the structure became significantly dense, hydration products notably increased, C-A-S-H gel dominated the structure, and system cementitious properties improved. With the addition of alkaline activator, as the curing time extended from 7 d to 28 d, all systems transformed from loose and porous to dense structures, hydration reactions became more complete, product types became richer, and, in addition to AFt, CaCO_3_ also appeared according to EDS (Table 6). These products jointly filled pores and improved structural stability, reflecting the time-dependent characteristics of Ca(OH)_2_ activation of CG [27,28]. Meanwhile, prolonged curing is favorable for complete hydration reactions.

##### Activation of CG Cementitious Properties by NaOH

**Figure 10:**
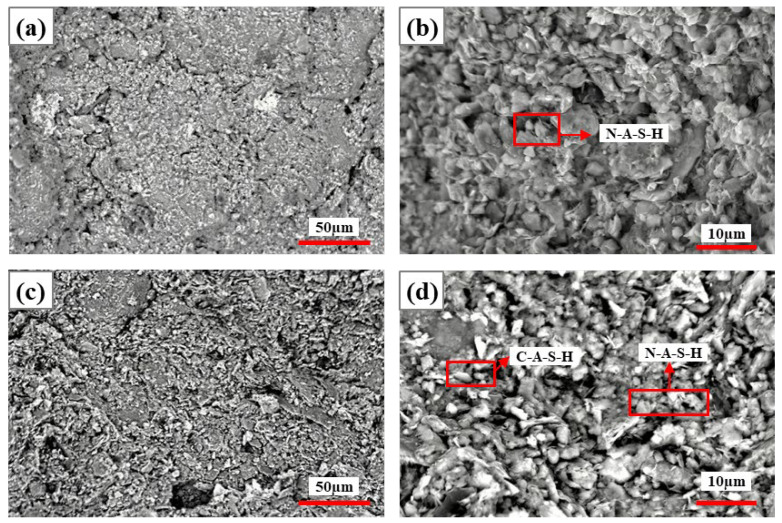
SEM images of specimens with 5% NaOH at 7 d and 28 d curing: (**a**,**b**) 7 d curing; (**c**,**d**) 28 d curing.

**Figure 11:**
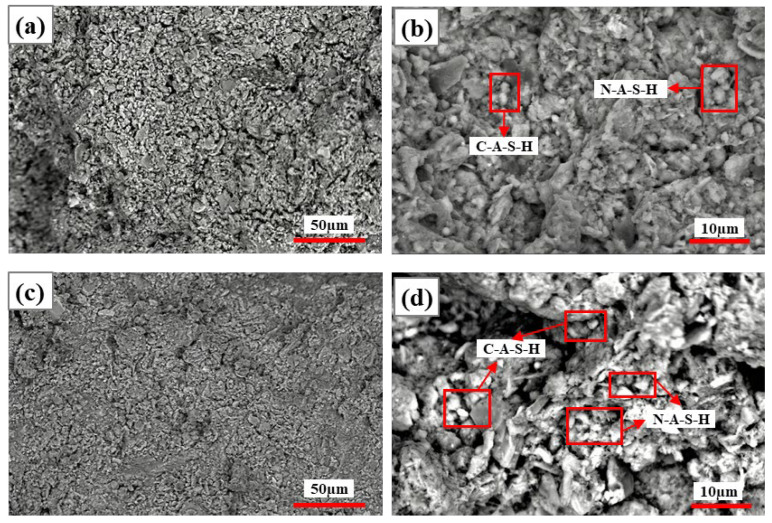
SEM images of specimens with 10% NaOH at 7 d and 28 d curing: (**a**,**b**) 7 d curing; (**c**,**d**) 28 d curing.

Figure 10 and Figure 11 show the SEM images of specimens at 5% and 10% NaOH dosage after 7 d and 28 d curing, respectively. At 5% NaOH dosage, at 7 d curing (Figure 10a,b), early hydration showed rough specimen surfaces, irregular particles, and many pores; at 28 d curing (Figure 10c,d), the structure was denser, large pores were reduced, and particles were tightly connected with network-like trends. At 5% NaOH dosage, according to EDS elemental ratios(Table 7), at 7 d curing (Figure 10b), mainly N-A-S-H gel formed with limited calcium participation; at 28 d curing (Figure 10d), the continued development of the gel network was observed morphologically, with Al-containing phases identified in local areas, while more continuous N-A-S-H networks also formed, and sodium-rich N-A-S-H gel also appeared.

As shown in Figure 11, at 10% NaOH dosage, at 7 d curing (Figure 11a,b), particle aggregates were relatively dispersed, with later-stage structure further densifying and particle boundaries becoming blurred. Early alkali-activation reactions were intense, generating large amounts of N-A-S-H gel with little calcium-based mineral participation; at 28 d curing (Figure 11c,d), N-A-S-H gel still dominated, with Na/Si ratio at some EDS measurement points decreasing (Table 8), gel structure transitioning to more stable networks, and silicon–aluminum polymerization degree increasing. In summary, with extended curing time, Na^+^ utilization became more complete, the microstructure progressively densified with extended curing time, suggesting continued reaction between the activator and CG particles. Meanwhile, the microstructure transformed from loose packing to dense, the element distribution became more uniform, and hydration product stability and cementing ability significantly improved.

Integrating the SEM-EDS analysis results, the microstructure evolution patterns of the three activator systems can be summarized as follows: (1) Extended curing time promotes structural densification in all systems, but the Na_2_SiO_3_ system achieves the highest degree of densification; (2) Alkali activators destroy CG crystal structures by providing an alkaline environment (OH^−^), causing Si-O-Si and Si-O-Al bond breakage, releasing soluble silicon–aluminum monomers (H_3_SiO_4_^−^, Al(OH)_4_^−^), which then cross-link and polymerize through Si-O-Al and Si-O-Si bonds to generate cementitious products (N-A-S-H, C-A-S-H, AFt, etc.), filling interparticle gaps and reducing porosity, ultimately improving material strength. In the Na_2_SiO_3_ system, N-A-S-H and C-A-S-H gels develop synergistically; in the Ca(OH)_2_ system, C-A-S-H dominates, accompanied by AFt; while the NaOH system is dominated by N-A-S-H; (3) 10% Na_2_SiO_3_ activator dosage combined with 28 d curing is the optimal condition for achieving sufficient reaction and structural densification.

## 4. Conclusions

(1) In terms of macroscopic properties, both activator chemistry and concentration exert strong control over the strength development of CG specimens. With increasing alkali activator dosage (5–12%), specimen compressive strength shows an increasing trend. Among them, Na_2_SiO_3_ has the best activation effect, with compressive strength reaching 6.4 MPa at 10% dosage after 28 d curing, a growth rate of 190.9%; Ca(OH)_2_ and NaOH have weaker activation effects, with strength growth rates of 69.55% and 62.27%, respectively, under the same conditions. The efficiency ranking of the three activators is as follows: Na_2_SiO_3_ > Ca(OH)_2_ > NaOH. Experimental results indicate that 10% Na_2_SiO_3_ dosage combined with 28 d curing is the optimal condition.

(2) Microstructure and phase evolution results further confirm the macroscopic property patterns. XRD analysis shows that Na_2_SiO_3_ sodium silicate activation leads to a greater reduction in crystalline mineral (quartz, kaolinite) peak intensities and more prominent amorphous gel formation compared to Ca(OH)_2_ and NaOH systems, with Ca(OH)_2_ additionally producing AFt.

(3) SEM-EDS analysis showed that at low activator dosage (5%), all systems exhibited loose, porous microstructures with substantial unreacted particles. At 10% dosage after 28 d curing, microstructural densification was significantly enhanced in all systems. In the sodium silicate system, co-existing N-A-S-H and C-A-S-H gels were observed with increased calcium content. In the Ca(OH)_2_ system, C-A-S-H and CaCO_3_ jointly filled interparticle pores. In the NaOH system, a more continuous N-A-S-H gel network developed compared to lower dosages and shorter curing durations. Na_2_SiO_3_, with its dual function of alkaline activation and active silicon supplementation, shows significant advantages in promoting CG activation.

It should be noted that the current study is limited to a pure CG system without supplementary cementitious materials, and the long-term durability and leaching behavior of the activated products remain to be investigated. Future work will focus on optimizing composite activator systems, evaluating the environmental safety of heavy metal immobilization, and scaling up the formulation for field application in mine backfill engineering.

## Figures and Tables

**Table 1:** Main chemical composition of CG samples.

Component	SiO_2_	Al_2_O_3_	Fe_2_O_3_	K_2_O	MgO	CaO	Na_2_O	SO_3_
Content (%)	62.55	22.94	5.158	3.151	1.90	1.31	1.22	0.206

**Table 2:** Mix proportions for CG cementitious performance tests.

Group	Sample ID	Alkali Activator Type	CG (g)	Activator Dosage (%)	W/B Ratio
1	0	-	300	0	0.3
2	Ca-5	Ca(OH)_2_	300	5.0	0.3
3	Ca-7	Ca(OH)_2_	300	7.0	0.3
4	Ca-10	Ca(OH)_2_	300	10.0	0.3
5	Ca-12	Ca(OH)_2_	300	12.0	0.3
6	Na-5	NaOH	300	5	0.3
7	Na-7	NaOH	300	7	0.3
8	Na-10	NaOH	300	10	0.3
9	Na-12	NaOH	300	12	0.3
10	Si-5	Na_2_SiO_3_	300	5	0.3
11	Si-7	Na_2_SiO_3_	300	7	0.3
12	Si-10	Na_2_SiO_3_	300	10	0.3
13	Si-12	Na_2_SiO_3_	300	12	0.3

**Table 3:** Based on the elemental composition derived from the EDS spectra in Figure 6.

Figure Number	Point	O	Na	Al	Si	S	Ca	Si/Al	Ca/Si	Na/Si
Figure 6b	N-A-S-H	49.48	2.35	10.73	21.14	-	1.99	1.97	0.09	0.11
	C-A-S-H	55.16	4.65	9.01	20.2	0.16	7.36	2.24	0.36	0.23
Figure 6d	N-A-S-H	48.77	5.51	12.75	25	-	0.58	1.96	0.02	0.22
	C-A-S-H	44.41	2.95	13.89	20.22	0.24	8.37	2.03	0.41	0.10

**Table 4:** Based on the elemental composition derived from the EDS spectra in Figure 7.

Figure Number	Point	O	Na	Al	Si	S	Ca	Si/Al	Ca/Si	Na/Si
Figure 7b	N-A-S-H	44.59	2.67	2.93	23.33	-	19.39	7.96	0.83	0.11
	C-A-S-H	52.63	1.16	4.19	16.06	-	22.49	3.83	1.40	0.07
Figure 7d	N-A-S-H	48.34	2.11	2.92	21.44	-	18.73	7.34	0.87	0.10
	C-A-S-H	53.85	1.8	2.83	18.81	0.4	16.17	6.65	0.86	0.10

**Table 5:** Based on the elemental composition derived from the EDS spectra in Figure 8.

Figure Number	Point	O	Na	Al	Si	S	Ca	Si/Al	Ca/Si	Na/Si
Figure 8b	C-A-S-H	55.48	0.44	7.38	14.96	0.06	17.35	2.03	1.16	0.03
Figure 8d	C-A-S-H	59.32	0.43	5.02	9.22	-	20.58	1.84	2.23	0.05
	AFt	52.95	0.46	6.5	18.72	0.17	15.78	2.88	0.84	0.02

**Table 6:** Based on the elemental composition derived from the EDS spectra in Figure 9.

Figure Number	Point	O	Na	Al	Si	S	Ca	Si/Al	Ca/Si	Na/Si
Figure 9b	C-A-S-H	48.16	0.09	12.68	23.92	0.22	9.29	1.89	0.39	0.00
Figure 9d	C-A-S-H	58.07	5.85	5.81	10.84	0.56	14.04	1.87	1.30	0.54
	AFt	55.63	0.52	8.51	11.71	1.23	12.49	1.38	1.07	0.04
	CaCO_3_	48.54	0.3	1.42	2.97	-	45.68	2.09	15.38	0.10

**Table 7:** Based on the elemental composition derived from the EDS spectra in Figure 10.

Figure Number	Point	O	Na	Al	Si	S	Ca	Si/Al	Ca/Si	Na/Si
Figure 10b	N-A-S-H	44.91	1.58	11.05	16.16	-	9.07	1.48	0.59	0.10
Figure 10d	C-A-S-H	51.98	0.33	13.57	18.87	-	7.36	1.39	0.39	0.02
	N-A-S-H	47.38	1.62	12.59	18.16	-	11.4	1.44	0.63	0.09

**Table 8:** Based on the elemental composition derived from the EDS spectra in Figure 11.

Figure Number	Point	O	Na	Al	Si	S	Ca	Si/Al	Ca/Si	Na/Si
Figure 11b	N-A-S-H	32.72	25.04	8.89	17.78	0.05	5.37	2.00	0.30	1.40
	C-A-S-H	46.98	2.12	14.32	17.55	0.13	10.42	1.22	0.59	0.12
Figure 11d	N-A-S-H	31.55	32.43	8.42	14.57	-	6.34	1.73	0.44	2.22
	C-A-S-H	49.65	3.03	13.85	18.26	0.16	8.65	1.32	0.47	0.16

## Data Availability

The original contributions presented in this study are included in the article. Further inquiries can be directed to the corresponding author.
